# Characterization and Comparison of Bioactive Polysaccharides from *Grifola frondosa* by HPSEC-MALLS-RID and Saccharide Mapping Based on HPAEC-PAD

**DOI:** 10.3390/polym15010208

**Published:** 2022-12-31

**Authors:** Baojie Zhu, Wenxia Zhang, Jing Zhao, Bilian Chen, Fei Liu, Shaoping Li

**Affiliations:** 1 Joint Laboratory of Chinese Herbal Glycoengineering and Testing Technology, University of Macau & National Glycoengineering Research Center, Macao SAR 999078, China; 2 State Key Laboratory of Quality Research in Chinese Medicine, Institute of Chinese Medical Sciences, University of Macau, Macao SAR 999078, China; 3 Zhejiang Institute of Food and Drug Control, Hangzhou 310052, China; 4 Shandong Academy of Pharmaceutical Sciences, Jinan 250101, China

**Keywords:** *Grifola frondosa* polysaccharides, saccharide mapping, HPSEC-MALLS-RID, HPAEC-PAD, immunomodulatory

## Abstract

*Grifola frondosa* polysaccharides (GFPs) from different regions in China were characterized and compared using HPSEC-MALLS-RID and saccharide mapping based on HPAEC-PAD analysis for achieving and improving its quality control. The results showed that HPSEC chromatograms and molecular weight distributions of GFPs were similar. The average contents of each polysaccharide fraction (Peaks 1, 2, and 3) showed that Peak 3 was the main component and much higher than the other two polysaccharide fractions, which also contained protein. The result of saccharide mapping showed that *α*-1,4-glycosidic, *β*-1,4-glycosidic and few *β*-1,3-glycosidic linkages were existed in GFPs. The similarity result showed that HPAEC-PAD fingerprints of the oligosaccharide fragments after hydrolysis by endoglycosidase were certainly different, especially *α*-amylase with a mean similar index of only 0.781 ± 0.207. The result of hierarchical cluster analysis (HCA) showed that different batches of GFPs from China can be divided into different clusters. Furthermore, immune-enhancing activity based on RAW 264.7 cells showed significant differences among different GFPs. Based on grey relational analysis (GRA), the fractions of Peak 3 were regarded as the major contributors to its immuno-enhancing activity in GFPs. Overall, the implications from these results were found to be stable, comprehensive, and valid for improving the quality control of GFPs.

## 1. Introduction

*Grifola frondosa*, a basidiomycete fungus belonging to the family of Grifolaceae and the order of Polyporales, has the homology of medicine and food due to its medical benefits, nutritional values, and unique flavors [1]. As one of the main active constituents of *Grifola frondosa*, *Grifola frondosa* polysaccharides (GFPs) have anti-tumor activity, immune regulation, liver protection, cardiovascular disease prevention, antihypertensive activities, etc. [2]. As complex mixtures, GFPs contain a variety of polysaccharide molecules, many of which play a key role in the biological effects of polysaccharides. However, few methods are available to analyze and characterize this polysaccharide. Therefore, developing a simple, efficient, and accurate method for the characterization and identification of GFPs can better enable its product development and quality control.

Saccharide mapping [3,4] based on endoglycosidase hydrolysis followed by kinds of saccharide analysis methods including high-performance liquid chromatography (HPLC) [5] and its variant size-exclusion chromatography (SEC) [6], polysaccharide analysis using carbohydrate gel electrophoresis (PACE) [7], high-performance thin layer chromatography (HPTLC) [8] has been successfully used for the quality control of polysaccharides from herbal medicine recent years, which possess various advantages of good repeatability, high resolution, and high throughput. However, these saccharide analysis methods have certain defects. For HPLC analysis, derivatization is often needed to improve the separation and detection sensitivity of saccharides due to high polarity and lack of chromophores [4]. Saccharide mapping based on HPTLC can usually be used to separate different types of monosaccharides and oligosaccharides with low degree of polymerization simply and quickly, however, it lacks high sensitivity. Although PACE has higher sensitivity for saccharide analysis and is more suitable for the separation of high-polymerization oligosaccharides, saccharides needed to be derivatized before analysis [9]. In addition to the above problems, saccharide mapping based on SEC, HPTLC and PACE also has a common disadvantage, which cannot realize the separation of oligosaccharide isomers. That means that some key information will be omitted when these techniques are used for saccharide mapping analysis and polysaccharide quality control, where it is entirely possible to obtain oligosaccharides with the same molecular weight but completely different structures.

High-performance anion-exchange chromatography with pulsed amperometric detection (HPAEC-PAD) is one of the most commonly used technologies for sugars and oligo- and polysaccharides analysis in biomass hydrolysates or food samples with high sensitivity, specificity, resolution, and without any derivatization [10]. Noteworthy results for the analysis of oligosaccharides encompass the analysis of xylooligosaccharides up to degree of polymerization 20 (DP 20) [11], glucooligosaccharides from amylopectin up to DP 50 [12], maltooligosaccharides up to DP 65 [13], as well as fructooligosaccharides up to DP 18 [14]. The more important thing is that it could realize the separation and detection of oligosaccharides isomers that possibly produced during polysaccharides hydrolysis [14,15]. Saccharide mapping based on HPAEC-PAD is believed to be beneficial for discriminating and improving the quality control of polysaccharides from traditional Chinese medicine.

In this study, polysaccharides from *G. frondosa* were characterized and compared by HPSEC-MALLS-RID and saccharide mapping based on HPAEC-PAD, and the immuno-enhancing effect was evaluated through NO release and phagocytic activity based on macrophages, which were helpful to obtain a better understanding of the structure–activity characters of polysaccharides from different regions and to improve their quality control of polysaccharides.

## 2. Materials and Methods

### 2.1. Materials and Reagents

Twelve batches of authentic *G. frondosa* (numbered 1–12), were collected from different regions of China (Table 1). All samples were identified by Professor Shaoping Li of the University of Macau, and the voucher samples were deposited in the Institute of Chinese Medical Sciences.

Monosaccharide standards (rhamnose, glucose, mannose, galactose, fucose, arabinose, xylose, ribose, glucuronic acid, and galacturonic acid) were acquired from Sigma-Aldrich (St. Louis, MO, USA). *α*-amylase (EC 3.2.1.1), *β*-glucanase (EC 3.2.1.6), cellulase (EC 3.2.1.4) were purchased from Megazyme (Wicklow, Ireland). Dulbecco’s Modified Eagle Medium (DMEM), fetal bovine serum (FBS), penicillin/streptomycin (P/S), and phosphate-buffered saline (PBS) were obtained from Gibco-Invitrogen (Paisley, Scotland, U.K.). Lipopolysaccharide (LPS), 3-(4,5-5dimethylthiazol-2-yl)-2,5-diphenyl tetrazolium bromide (MTT), Griess reagent, fluorescein isothiocyanate-conjugated dextran (FITC-Dextran 40000), and trifluoroacetic acid (TFA) were purchased from Sigma-Aldrich (St. Louis, MO., USA.). Sodium hydroxide standard solution (1M) was purchased from Aladdin Reagent Co., Ltd. (Shanghai, China). Deionized water was prepared by a Millipore Milli-Q Plus system (Millipore, Bedford, MA, USA). All the other chemicals and solvents were of analytical grade.

### 2.2. Preparation of Polysaccharides from G. frondosa

The samples were vacuum dried at 60 °C for 24 h and pulverized. To remove pigments, lipids, and other small molecules, the powder of *G. frondosa* was refluxed with 80% ethanol firstly at 100 °C for 2 h at the solid to liquid ratio of 1:20. Then, the residue was refluxed with deionized water at 100 °C for 2 h at the same solid to liquid ratio of 1: 20. After cooling to the room temperature, the supernatant was obtained by centrifugation at 4000 *g* for 20 min. Subsequently, the collected supernatant was concentrated to an appropriate volume under vacuum at 60 °C, and then 4 times the volume of 95% ethanol was added for precipitation. After standing overnight at 4 °C, it was centrifuged at 4000 *g* for 15 min to obtain polysaccharide precipitation. The polysaccharide precipitation was redissolved with distilled water, and then ultrafiltration was performed with 3 kDa ultrafiltration cube (Millipore, Billerica, MA, USA) at 2000 *g* to further remove the low molecular weight compounds. After ultrafiltration, the concentrated solution was freeze-dried to obtain water-soluble GFPs.

### 2.3. Monosaccharide Composition of GFPs

The complete acid hydrolysis condition was modified slightly according to previous report method [16]. Briefly, the lyophilized GFPs were hydrolyzed by TFA at 100 °C for 4 h. The final concentration of GFPs solution and TFA was 2 mg·mL^−1^ and 2 mol·L^−1^. After cooling to room temperature, hydrolysates were centrifuged at 4000× *g* for 5 min, and then dried under nitrogen. For removing TFA completely, appropriate amount of ethanol was used to dissolve the dried hydrolysates and then dried under nitrogen again.

### 2.4. Partial Acid Hydrolysis of GFPs

Partial acid hydrolysis of GFPs was applied based on description of report method [17] with slight modifications. In brief, the lyophilized GFPs were hydrolyzed by TFA at 80 °C for 2 h. The final concentration of GFPs solutions and TFA was 2 mg·mL^−1^ and 1 mol·L^−1^. Finally, all the samples were dried under nitrogen, and ethanol was also used to remove TFA completely as described above. GFPs solutions without TFA addition that treated as the same steps as above was used as the blank control.

### 2.5. Enzymatic Hydrolysis of GFPs

For enzymatic hydrolysis, the GFPs solutions (2 mg·mL^−1^ 200μL) were mixed with equal volume enzyme solution (40 U·mL^−1^) and digested at incubated at 40 °C for 12 h. Then, the mixtures were inactivated at 80 °C for 10 min to denature the enzymes. After centrifugation at 10,000× *g* for 10 min, the supernatants were collected and freeze-dried for the following analysis. The selected enzymes for hydrolysis were cellulase, *β*-1,3-glucanase, *α*-amylase according to related glycosidic bonds which have been previously reported [18]. GFPs solution without enzymes that treated as the same steps as above was used as the blank control.

### 2.6. HPSEC-MALLS-RID Analysis

High-performance size-exclusion chromatography coupled with multi-angle laser light scattering/refractive index detector (HPSEC-MALLS-RID) is more and more widely used to evaluate polysaccharides due to its strong ability in obtaining information of polysaccharides such as concentration, relative molecular weight, conformation characterization, etc. [19,20]. The system was equipped with an Agilent 1260 series LC/DAD system (Agilent Technologies, Palo Alto, CA, USA) coupled with a Multi-Angle Light Laser Scattering Detectors (MALLS, Wyatt Technology Co., Santa Barbara, CA, USA) and an Optilab rEX refractometer (RID, Wyatt Technology Co., Santa Barbara, CA, USA). The polysaccharides were separated with TSK-GEL G5000PW_XL_ (300 mm × 7.8 mm, 10 μm) and TSK-GEL G3000PW_XL_ (300 mm × 7.8 mm, 7 μm) column. The MALLS instrument was equipped with a He-Ne laser (λ = 658 nm) and Zimm method was applied to conduct the molecular weight calculation [21]. According to the responses of the RID, the universal refractive index increment (*dn*/*dc*) value of 0.150 mL·g^−1^ was used to calculate the polysaccharides concentration. NaCl aqueous solution (9%, *w*/*v*) was used as mobile phase with flow rate of 0.5 mL·min^−1^. The dried enzymatic hydrolysates were dissolved by 200 μL mobile phase and each GFPs (~2 mg) was dissolved in 1.0 mL 0.9% NaCl aqueous solution with the final concentration of 2 mg·mL^−1^. All samples were centrifuged at 12,000× *g* for 10 min at 10 °C and the supernatants were collected for HPAEC-MALLS-RID analysis. The injection volume was 100 μL. For data acquisition and analysis, the Astra software (version 6.0.2, Wyatt Technology Co., Santa Barbara, CA, USA) was applied.

### 2.7. HPAEC-PAD Analysis

The Dionex ICS-5000^+^ Ion Chromatography System equipped with an analytical gradient pump, an eluent degas module and a pulsed amperometric detection (PAD) with a gold working electrode and a PdH reference electrode. The separation was performed on CarboPac PA200 column (3 mm × 250 mm) with a guard column CarboPac PA200 (3 mm × 50 mm) at 30 °C. Chromeleon^®^ (7.0) software was used for data processing.

For analysis of the monosaccharide composition, each dried sample was dissolved by 1 mL deionized water and diluted 20 times and filtered by 0.22 μm membrane before analysis. The analyses were performed in gradient mode of 2 mmol·L^−1^ sodium hydroxide (A) and 250 mmol·L^−1^ sodium acetate-2 mmol·L^−1^ sodium hydroxide (B) (100% A 0–10 min, 100–50% A 10–25 min). At the end of analysis, the column was regenerated with 200 mmol·L^−1^ sodium hydroxide for 10 min.

For the separation of oligosaccharides from GFPs hydrolysates by different hydrolysis methods, 100 mmol·L^−1^ sodium hydroxide (eluent A) and 500 mmol·L^−1^ sodium acetate-100 mmol·L^−1^ sodium hydroxide (eluent B) was performed starting with 100% eluent A at 0 min with a linear increase in eluent B to reach 50% within 40 min. Before each injection, column equilibration was performed by maintaining starting conditions for at least 10 min. Each sample was dissolved by 1 mL deionized water and filtered with 0.22 μm membrane before analysis. The injection volume was 2.5 μL and the flow rate was maintained at 0.5 mL·min^−1^.

### 2.8. Effects of GFPs on Macrophage Functions

#### 2.8.1. Cell Culture

Murine macrophages RAW264.7 cells were obtained from American Type Culture Collection (ATCC, Rockville, MD, USA) and cultured in Dulbecco’s Modified Eagle Medium (DMEM) supplemented with 10% Fetal Bovine Serum (FBS), 1% penicillin/streptomycin (P/S) at 37 °C in a humidified 5% CO_2_. Cells were harvested for experiments in the exponential growth period.

#### 2.8.2. Cell Viability Assay

RAW 264.7 cells (5 × 10^3^ cells/well) were cultured in 96-well microplates overnight and treated with serial concentrations (10~160 μg·mL^−1^) of GFPs for 24 h. Blank control and positive control group were prepared by replacing GFPs solution with equal volume of culture medium and LPS at work concentration of 0.4 μg·mL^−1^. Then, 10 μL of 5 mg·mL^−1^ MTT solution in PBS (pH 7.4) was added to each well and incubated for additional 4 h in the dark. After discarding the medium, 100 μL DMSO was added to dissolve the formazan crystals in each well. Finally, the absorbance was detected at 570 nm using a microplate reader (1420 Multilabel counter victor, Perkin-Elmer, Waltham, MA, USA). The percentage of cell viability was expressed as the ratio of absorbance values between samples and blank control groups.

#### 2.8.3. Nitric Oxide Assay

Nitric oxide (NO) releases were measured by the Griess reaction [22]. RAW 264.7 cells (5 × 10^4^ cells/well) were cultured in 96-well microplates overnight and treated with serial concentrations (10~160 μg·mL^−1^) of GFPs for 24 h. Blank control and positive control group were prepared by replacing GFPs solution with equal volume of culture medium and LPS at work concentration of 0.4 μg·mL^−1^. Then, 75 μL of cell culture supernatant was taken and mixed with an equal volume of Griess reagent at room temperature for 15 min. The absorbance was detected at 540 nm with microplate reader. The NO levels were expressed as the percentage of absorbance values between samples and LPS-treated groups.

#### 2.8.4. Phagocytic Activity Test

RAW 264.7 cells (2 × 10^5^ cells/well) were cultured in 24-well microplates overnight and treated with GFPs at 40 μg·mL^−1^ for 18 h. Blank control and positive control group were prepared by replacing GFPs solution with equal volume of culture medium and LPS at work concentration of 0.4 μg·mL^−1^. Subsequently, cells were cultured with medium containing FITC-dextran (0.1 mg·mL^−1^) for another 1 h in the dark. Then, cells were washed three times with 1 X PBS and the cell suspension was collected and analyzed by a BD Accuri™ C6 Cytometer (BD Biosciences, San Jose, CA, USA). For each sample, 10,000 cells were recorded. The phagocytosis was represented as ratio of phagocytic rate between samples and LPS-treated groups under the support of the data analysis tool, FlowJo software (TreeStar, San Carlos, CA, USA).

#### 2.8.5. Determination of Endotoxin Contamination

The endotoxin concentration in the GFPs were detected by using Limulus Amebocyte Lysate assay (Lonza, Walkersville, Md., USA) with a Glucashield reconstitution buffer formulated to block interference of (1,3)-*β*-D-glucans (Associates of Cape Cod, East Falmouth, MA, USA) according to the manufacturer’s protocol. Escherichia coli O113:H10 endotoxin (Associates of Cape Cod) was used as endotoxin standard, and the concentration of endotoxin in the test samples was calculated from the calibration curves constructed by series concentrations of endotoxin standard. The results showed that GFPs contained endotoxin less than 0.01 EU·μg^−1^, which could exclude endotoxin contamination in GFPs.

### 2.9. Data Analysis and Multivariate Analysis

Analyses were performed in triplicates, and results were expressed as means ± standard deviations. For multiple group comparisons, one-way analysis of variance (ANOVA) was conducted by Duncan’s post hoc test and applied for determining the significance (*p* < 0.05) differences. The similarities of the tested samples and the simulative mean chromatogram were calculated and produced by the professional software “Similarity Evaluation System for Chromatographic Fingerprint of Traditional Chinese Medicine (TCM)”. Other statistical analysis was carried out using Microsoft 2016 package and SPSS software (version 25.0, SPSS Inc., USA.), including hierarchical cluster analysis (HCA) for similarity display and grey relational analysis (GRA) for fingerprint-activity relationships between HPSEC fingerprints and effects of GFPs on macrophages.

## 3. Results and Discussion

### 3.1. Molecular Weight, Monosaccharides Composition and Content of Polysaccharides from G. frondosa

**Figure 1:**
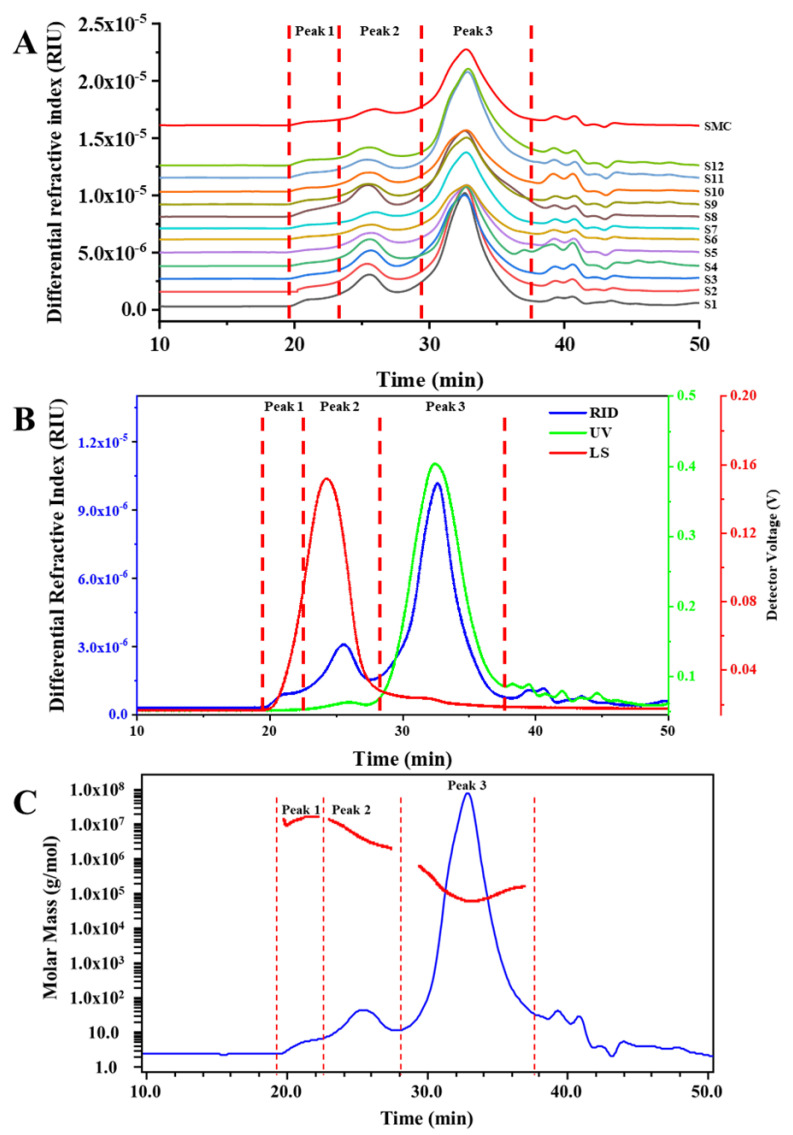
Comparison of HPSEC-MALLS-RID spectra of polysaccharides from *G. frondosa*. (**A**) HPSEC-RID fingerprints; (**B**) Typical HPSEC-MALLS-RID chromatogram; (**C**) HPSEC-RID profile with molecular weight distribution.

For polysaccharides from natural products, HPSEC chromatograms and molecular weights are essential for its discrimination and improvement of quality control. [9,23]. Therefore, the molecular weight (Mw), polydispersity index (Mw/Mn) and content of different polysaccharides fractions in *G. frondosa* were obtained by HPSEC-MALLS-RID. As showed in Figure 1, there were three main polysaccharides fractions (Peak 1~3) in GFPs, and HPSEC chromatograms of *G. frondosa* from different regions in China were similar. However, Mw, Mw/Mn and content of same polysaccharides fractions in GFPs from different regions were different (Table 1). The molecular weight was ranging from 5.00 × 10^6^ to 8.71 × 10^6^ Da (Peak 1), 2.63 × 10^6^ to 5.64 × 10^6^ Da (Peak 2), and 1.01 × 10^5^ to 1.51 × 10^5^ Da (Peak 3), respectively. On the other hand, the Mw/Mn of Peak 1 was very close to 1.00 compared with the other two polysaccharides fractions, which indicated that it had good homogeneity, but its content is much lower than that of the other two components. The content results of different polysaccharides fractions in GFPs showed that Peak 3 was the main component and much higher than the other two polysaccharide fractions, which also contained protein through Ultraviolet (UV) absorbance detected at UV 280 nm. In addition, the similarity of the HPSEC fingerprints was calculated, and the correlation coefficients (r) of 12 batches of samples were listed in Table 2. The results showed that all r values of HPSEC chromatograms were larger than 0.980.

The monosaccharide composition of GFPs from different regions in China was shown in Table S1. The result showed that glucose was the predominant monosaccharides in all samples expect S11 and S12, which is basically similar to that reported by other research articles [24,25]. However, the molar ratios of glucose in each sample varied much, ranging from 1.84 to 11.76. The second major monosaccharides were galactose, mannose and fucose with molar ratios of 1.85–2.37, 1.31–1.75 and 1.00, respectively. Xylose (<0.15) was present in all samples with relatively low levels.

### 3.2. Saccharide Mapping based HPSEC-DAD-RID

**Figure 2:**
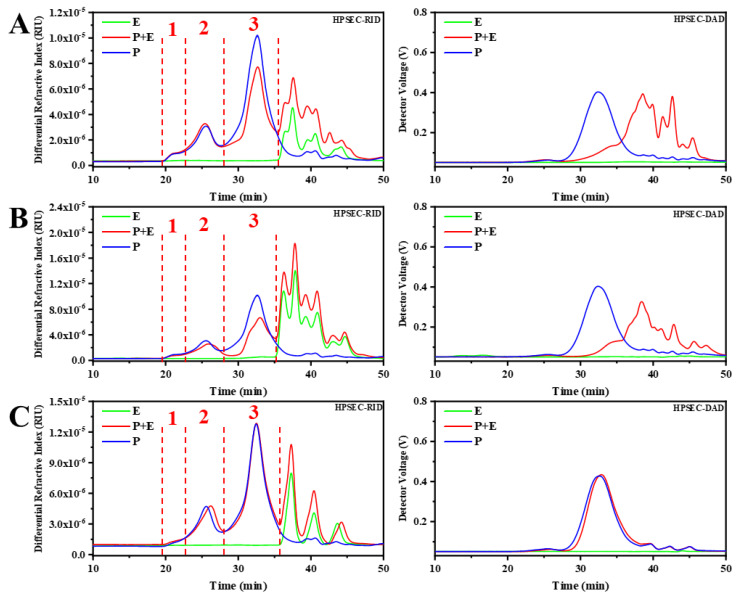
HPSEC-RID (**left**) and HPSEC-DAD (**right**) chromatography profiles of GFPs treated with (P+E) or without (P) selected enzymes (E). (**A**): *α*-amylase, (**B**): cellulase, (**C**): *β*-1,3-glucanase.

Previous studies have shown that polysaccharides from *G. frondosa* contain *α*-1, 4-glycosidic linkage [26,27], *β*-1,4-glycosidic linkage [28], and *β*-1,3-glycosidic linkage [24]. Therefore, *α*-amylase, cellulase and *β*-1,3-glucanase were selected for depolymerization of polysaccharides in *G. frondosa*. HPSEC-RID and HPSEC-DAD profiles of GFPs before and after digestion by selected enzymes were shown in Figure 2. The results of HPSEC-RID showed that *α*-amylase and cellulase can hydrolyze GFPs corresponding to Peak 2 and Peak 3, especially Peak 3. However, the HPSEC-RID profiles of polysaccharides from *G. frondosa* have only minor changes after hydrolyzing by *β*-1,3-glucanase, which means the content of polysaccharides fraction with *β*-1,3-glycosidic linkage was very low. In addition, the HPSEC-DAD profiles showed that the UV response was significantly reduced after hydrolysis with *α*-amylase and cellulase. This result showed that the polysaccharides fractions which contain *α*-1,4-glycosidic linkage and *β*-1,4-glycosidic linkage in GFPs may be the polysaccharides-protein complex.

### 3.3. Saccharide Mapping Based on HPAEC-PAD

#### 3.3.1. Partial Acid Hydrolysis Fingerprints of GFPs

**Figure 3:**
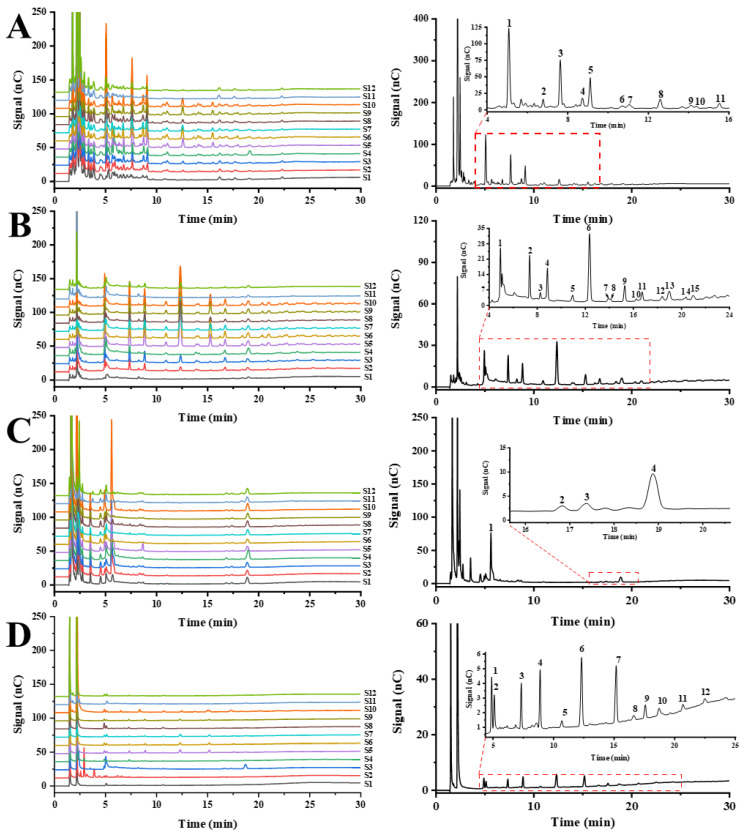
The HPAEC-PAD fingerprints (**left**) and the simulative mean chromatogram (**right**) of different hydrolysates from GFPs after partial acidic hydrolysis by TFA (**A**) and enzymatic hydrolysis by *α*-amylase (**B**), cellulase (**C**) and *β*-1,3-glucanase **(D**).

The chemical structures of homo- and heteropolysaccharides can generally be elucidated by partial acid hydrolysis under the support analysis of the resulting oligosaccharides. Therefore, the partial acid hydrolysis was performed to degrade the GFPs for establishing the understanding of the chemical structures. As a result, the HPAEC-PAD fingerprints of the 12 samples after partial acid hydrolysis were established in Figure 3A. The similarity analysis showed that the average correlation coefficient of each chromatogram to their simulative mean chromatogram was 0.967 ± 0.044 (*n* = 12), which supported the fact that partial acid fingerprint of polysaccharides from 12 batches of *G. frondosa* were very similar except S11 and S12 (Table 2). The result showed that polysaccharides from S11 and S12 were different from each other, especially the polysaccharides from S11 with the correlation coefficient is 0.846. Therefore, partial acid hydrolysis profiles are helpful to improve the quality control of polysaccharides from *G. frondosa*. However, acid hydrolysis lacks specificity, in consequence, enzymatic hydrolysis based on chemical structural characters of polysaccharides was further investigated.

#### 3.3.2. Enzymatic Hydrolysis Fingerprints of GFPs

Enzymatic hydrolysis is a mild digestion approach with such advantages as high selectivity, high efficacy, and environmental protection, which is crucial for saccharide mapping to differentiate and characterize the polysaccharides from natural resources [3]. In this study, *α*-amylase, cellulase and *β*-1,3-glucanase were selected for construction of the enzymatic fingerprints of polysaccharides in *G. frondosa*.

HPAEC-PAD fingerprints of different hydrolysates from GFPs after hydrolyzing by *α*-amylase, cellulase and *β*-1, 3-glucanase were shown in Figure 3B–D. The results showed that GFPs can be hydrolyzed by *α*-amylase, cellulase and *β*-1,3-glucanase to a certain extent, which further prove the existence of *α*-1, 4-glycosidic linkage, *β*-1,4-glycosidic linkage, and *β*-1,3-glycosidic linkage. The chromatograph of blank control group showed that none of the sugars existed in the samples before enzymatic hydrolysis (Figure S1). Most notable, though, is that the oligosaccharide fragments from diffident GFPs after depolymerizing by the same select enzyme had some differences, especially in terms of content. These discrepancies mostly resulted from different content of polysaccharides fractions with the same glycosidic linkage in GFPs, which will have a far-reaching impact on its quality control. Moreover, the similarities of their entire chromatographic patterns were evaluated, and the correlation coefficient values were summarized in Table 2. The results showed that the average correlation coefficient value of each HPAEC-PAD chromatogram to their simulative mean chromatogram was below 0.950, especially HPAEC-PAD fingerprint of hydrolysates after hydrolyzing by *α*-amylase, which was only 0.781 ± 0.207. Therefore, enzymatic hydrolysis profiles are better helpful to improve the quality control of polysaccharides from *G. frondosa* compared to partial acid hydrolysis.

#### 3.3.3. HCA of the Saccharide Mapping Based on HPAEC-PAD Chromatogram and Common Peaks

**Figure 4:**
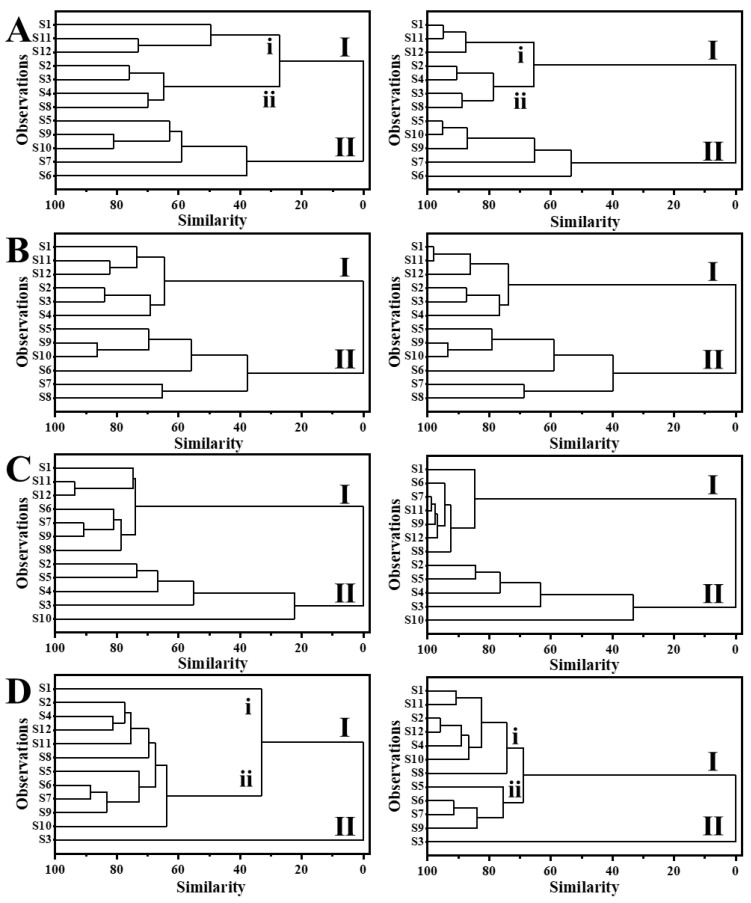
Dendrogram of hierarchical cluster analysis of GFPs based on HPAEC-PAD chromatograms (**left**) and relative area of common peaks (**right**). Different hydrolysates from GFPs were obtained by partial acid hydrolysis (**A**) and enzymatical hydrolysis with *α*-amylase(**B**), cellulase (**C**), and *β*-1,3-glucanase (**D**).

To quantify the similarity of the samples and visually compare the differences between different samples, hierarchical cluster analysis (HCA) was performed based on entire HPAEC-PAD chromatographic patterns and common peaks (Figure 4). The results showed that 12 batches of GFPs were divided into two clusters of I and II whether based on entire HPAEC-PAD chromatographic patterns or common peaks. For partial acid hydrolysis, cluster I was further divided into subcategories of i and ii, and the results based on entire chromatographic patterns and common peaks are completely consistent. For enzymatic hydrolysis with *α*-amylase and cellulase, HCA based on entire HPAEC-PAD chromatograms and common peaks also showed consistent results. Interestingly, according to partial acid hydrolysates and *α*-amylase hydrolysates, the samples covered in the same cluster were similar, however, the result of *α*-amylase hydrolysates with higher stability and repeatability because of the enzyme specificity. For the HCA results of enzymatic hydrolysates with *β*-1,3-glucanase, which are slightly different based on common peaks showed only one sample (S1) was divided into i group, which is different from the HCA result based on the entire HPAEC-PAD chromatograms. Therefore, based on the common peaks of different hydrolysates, the differences among different samples can be visually compared and quantified, which better realizes the quality control of GFPs.

### 3.4. Effects of Polysaccharides from G. frondosa (GFPs) on Macrophage Functions

For polysaccharides from natural products, there is a close connection between bioactivities and their chemical properties, such as molecular size, types and ratios of constituent monosaccharides, and features of glycosidic linkages [29]. In this study, a systematic investigation of chemical properties was conducted on GFPs, including molecular weight distributions, monosaccharides compositions, HPAEC-PAD fingerprints of their partial acid and enzymatic hydrolysates. Polysaccharides are one of the most important bioactive compounds in *G. frondosa*, especially in immunomodulation activity [18], as macrophages are indispensable cells playing a central role in immune system and participating in a wide variety of biological processes. In this study, to better understand the structure-activity relationship of GFPs, RAW 264.7 cells, a murine macrophage-like cell line, were used to evaluate the immunomodulatory effect of GFPs in vitro.

#### 3.4.1. Effects of GFPs on Cell Proliferation

Cell viability of RAW 264.7 cells treated with series concentrations (10 ~ 160 μg·mL^−1^) of GFPs or 0.4 μg·mL^−1^ LPS were evaluated by MTT assay. As shown in Table S2, only several GFPs showed slight proliferation-stimulating activity at low concentration (10 ~ 20 μg·mL^−1^), whereas all GFPs at high concentration (160 μg·mL^−1^) performed cytotoxicity significantly (*p* < 0.05). IC10 was calculated to assess the minimal concentration of the GFPs that cause initial cytotoxicity. The relatively low IC10 value for S4 and S6 indicated a higher cytotoxicity of these two GFPs to the cells. Apart from the glycosidic chains, other impurities such as proteins still existed in GFPs, which are possibly toxic to cells.

#### 3.4.2. Effects of GFPs on Cell Proliferation

**Figure 5:**
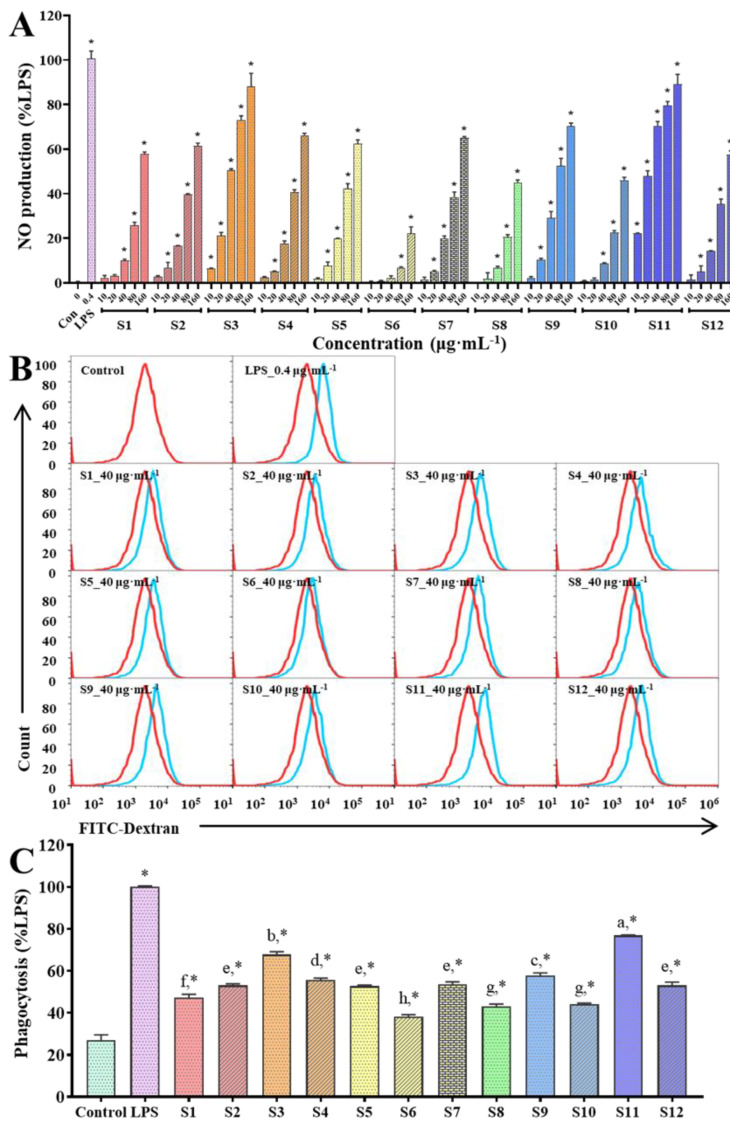
Immune-enhancing activity of GFPs evaluated via RAW 264.7 macrophages. (**A**) Nitric oxide (NO) production from macrophages treated with different concentrations of GFPs; Typical flow cytometric profiles (**B**) and histograms (**C**) of phagocytosis activity of macrophages treated with GFPs at concentration of 40 μg·mL^−1^. The percentage of NO production and phagocytosis was calculated relative to the lipopolysaccharide (LPS) -treated groups. Data were expressed as mean ± SD of three independent experiments. *, *p* < 0.05 was labeled versus vehicle control. Different letters indicated significant difference between GFPs-treated group (*p* < 0.05).

Nitric oxide (NO), an essential signaling molecule, acts as a host defense effector in the immune system and could also act as a cytotoxic agent in pathological processes [30]. Therefore, nitric oxide was chosen for evaluation of macrophages activation in vitro. Nitric oxide could be transformed into nitrite and dissolved in the supernatant, which could be further detected by the Griess method [22]. The effect of GFPs on stimulating RAW 264.7 cells releasing NO was shown in Figure 5A. The results presented that all GFPs had significantly increased effects on NO production of RAW 264.7 cells (*p* < 0.05), and GFPs from S11 and S3 had most potent ability to stimulate the production of NO from macrophages.

#### 3.4.3. Effects of GFPs on Phagocytosis of Macrophages

As the most pronounced character of macrophages, the improvement of phagocytosis is the most straightforward evidence of macrophage activation. Thus, the effects of GFPs on phagocytic activity of macrophages was evaluated by uptake of FITC-Dextran using flow cytometry analysis. Due to obvious difference presented by GFPs in results of NO production under 40 μg·mL^−1^ treatment, the concentration of 40 μg·mL^−1^ was chosen to conduct this experiment to better explain the difference between the immune-stimulating ability of GFPs. LPS treatment was applied as a positive control. As shown in Figure 5B,C, all GFPs showed significant promotion effect on phagocytic activity compared to vehicle group (*p* < 0.05). The similar situation was also observed that GFPs from S11 had the strongest phagocytosis-promoting effect of macrophages, which almost reached 3-fold increase compared with the untreated group. This result further illustrated the close relationship between immune-enhancing activity and polysaccharides fractions with *α*-1,4-glycosidic, *β*-1,4-glycosidic and *β*-1,3-glycosidic bonds of GFPs.

### 3.5. HPSEC Fingerprint-Immune Activity Relationship

**Figure 6:**
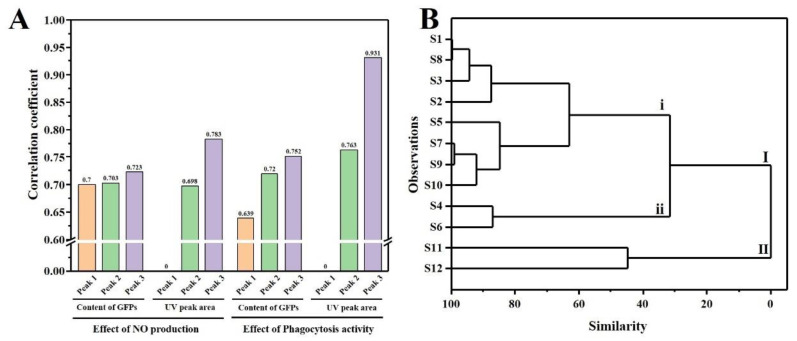
The correlation coefficient between the common characteristic peaks and the efficacy of GFPs (**A**) and dendrogram of hierarchical cluster analysis of GFPs based on the RID and UV peak area of Peak 3 (**B**).

Grey relational analysis (GRA) method was used to establish quantitative fingerprint-activity relationship model built with 3 common peaks of HPSEC-RID fingerprint and 2 common peaks of HPSEC-DAD fingerprint with effects of GFPs on NO production and phagocytosis of macrophages. As is shown in Figure 6A, no matter the analysis results based on HPSEC-RID or HPSEC-DAD, Peak 3 is the main contributor to the immune activity of GFPs. For further characterizing the difference of GFPs from different regions in China, as the main active component, the RID and UV peak area of Peak 3 were used for hierarchical cluster analysis (Figure 6B). The result showed that 12 batches of GFPs were divided into two clusters of I and II. The samples from Hunan province (S11 and S12) were divided into cluster II, and other samples from Zhejiang, Fujian and Hebei province were divided into cluster I. In addition, although cluster I was further divided into subcategories of i and ii, only two samples (S4 and S6) from Zhejiang province were included.

## 4. Conclusions

In the present study, an evaluation system including HPSEC-MALLS-RID, saccharide mapping based on HPAEC-PAD and were successfully used for comparison of polysaccharides in *G. frondosa* from different regions in China. The results showed that differences were observed between GFPs from the aspects of molecular weight, contents of different fractions, and immuno-enhancing activities evaluated via macrophages. The result of saccharide mapping based on HPAEC-PAD showed that *α*-1,4-glycosidic, *β*-1,4-glycosidic and few *β*-1,3-glycosidic linkages were existed in GFPs. Furthermore, hydrolysates of GFPs after hydrolysis by different endoglycosidase also showed certain differences, especially the hydrolysates obtained from *α*-amylase with a mean similar index of only 0.781 ± 0.207. The further HCA analysis showed that different batches of GFPs from China can be divided into different clusters no matter based on the entire HPAEC-PAD chromatograms or based on common peaks. The results were helpful to improve the quality control of polysaccharides from different regions, which also has the great potential for quality evaluation of polysaccharides from other herbal medicine. In addition, the combined comparison of the fingerprint-activity relationships between HPSEC fingerprints and bioactivities of polysaccharides from *G. frondosa* is further helpful to improve their pharmacological activity-based quality control.

## Figures and Tables

**Table 1:** Molecular weight and content of different polysaccharides fractions in GFPs from different regions in China.

Code	Regions		Peak 1			Peak 2			Peak 3	
		Mw×10^6^ /Da	Mw/Mn	Content/%	Mw×10^6^ /Da	Mw/Mn	Content/%	Mw×10^5^/ Da	Mw/Mn	Content/%
S1	Zhejiang Province	5.06	1.06	1.50	3.88	1.41	14.75	1.22	1.77	62.75
S2	Zhejiang Province	5.97	1.08	1.75	3.70	1.32	13.25	1.28	1.72	59.90
S3	Zhejiang Province	6.78	1.02	1.46	3.29	1.38	12.60	1.24	1.64	53.70
S4	Zhejiang Province	7.35	1.04	0.65	3.72	1.36	11.86	1.26	1.71	44.14
S5	Zhejiang Province	7.01	1.02	1.00	3.11	1.49	9.90	1.08	1.52	42.55
S6	Zhejiang Province	6.80	1.05	1.15	3.18	1.47	7.30	1.23	1.57	36.70
S7	Zhejiang Province	6.87	1.03	1.65	2.63	1.59	7.85	1.01	1.55	49.05
S8	Hebei Province	5.51	1.09	1.19	3.69	1.59	18.86	1.11	1.70	57.74
S9	Fujian Province	8.71	1.03	1.50	3.84	1.42	10.30	1.39	1.61	43.50
S10	Fujian Province	5.00	1.09	1.30	3.26	1.36	9.50	1.11	1.60	43.60
S11	Hunan Province	5.48	1.02	1.90	5.64	1.38	9.15	1.51	1.66	66.00
S12	Hunan Province	8.49	1.06	2.10	4.17	1.36	9.30	1.21	1.61	61.40

Note: The content here refers to the proportion of different polysaccharides fractions in GFP.

**Table 2:** The correlation coefficients (*r*) of GFPs fingerprints by HPSEC and hydrolysates fingerprints by HPAEC-PAD.

Code	HPSEC	HPAEC-PAD			
	GFPs	Partial acid	*α*-amylase	Cellulase	*β*-1,3-glucanase
S1	0.990	0.954	0.400	0.843	0.912
S2	0.994	0.992	0.754	0.968	0.957
S3	0.990	0.996	0.835	0.939	0.985
S4	0.981	0.995	0.651	0.980	0.999
S5	0.999	0.981	0.934	0.955	0.998
S6	0.996	0.954	0.924	0.925	0.992
S7	0.995	0.989	0.981	0.991	0.781
S8	0.990	0.996	0.969	0.970	0.998
S9	0.997	0.989	0.922	0.994	0.954
S10	0.992	0.984	0.912	0.980	0.978
S11	0.991	0.846	0.391	0.699	0.713

Note: Values in each column represent the correlation coefficients (r) of corresponding fingerprint.

## Data Availability

The data presented in this study are available on request from the corresponding author.

## Supplementary Materials

The following supporting information can be downloaded at: https://www.mdpi.com/article/10.3390/polym15010208/s1, Figure S1: The HPAEC-PAD profiles of different hydrolysates from GFPs before hydrolysis; Table S1: Monosaccharide composition of the polysaccharides from *G. frondose*; Table S2: Effects of polysaccharides from *G. frondose* (GFPs) on RAW 264.7 cells viability.
