# Supplementary material for: Characterization and Comparison of Bioactive Polysaccharides from Grifola frondosa by HPSEC-MALLS-RID and Saccharide Mapping Based on HPAEC-PAD
Source: Polymers (Basel). 2022 Dec 31;15(1):208. doi: 10.3390/polym15010208 (PMC9824690; doi:10.3390/polym15010208)
Supplement: Supplementary file 1 [file polymers-15-00208-s001.zip › polymers-2106851-supplementary.pdf]

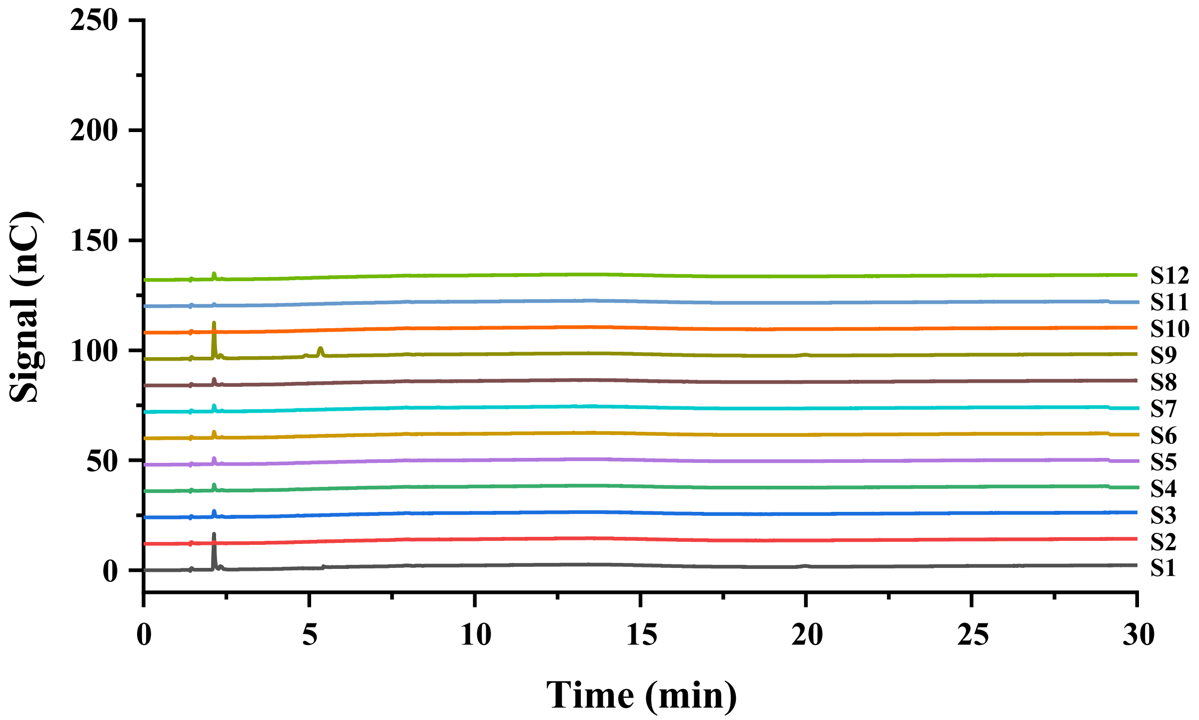

Figure S1. The HPAEC-PAD profiles of different hydrolysates from GFPs before hydrolysis

Table S1 Monosaccharide composition of the polysaccharides from *G. frondose*

Code	Molar ratios				
	Fucose	Galactose	Glucose	Xylose	Mannose
S1	1.00	2.34	2.80	0.03	1.37
S2	1.00	2.26	3.28	0.03	1.39
S3	1.00	2.17	4.07	0.02	1.46
S4	1.00	2.23	4.22	0.02	1.53
S5	1.00	2.13	8.95	0.10	1.75
S6	1.00	2.23	11.76	0.09	1.74
S7	1.00	1.94	6.97	0.12	1.58
S8	1.00	2.37	4.81	0.02	1.38
S9	1.00	2.27	8.28	0.06	1.65
S10	1.00	2.18	8.82	0.06	1.68
S11	1.00	1.95	1.92	0.03	1.40
S12	1.00	1.85	1.84	0.02	1.31

Table S2 Effects of polysaccharides from *G. frondose* (GFPs) on RAW 264.7 cells viability

Code	Cell viability (% control)					IC_{10} (μg·mL^{-1})
	10 μg·mL^{-1}	20 μg·mL^{-1}	40 μg·mL^{-1}	80 μg·mL^{-1}	160 μg·mL^{-1}	
S1	113.05 ± 5.34^{ab,***}	108.18 ± 5.53^{a,*}	101.81 ± 0.46^{a}	92.36 ± 0.41^{a}	78.51 ± 3.49^{ab,***}	108.60
S2	107.06 ± 5.31^{bcd}	105.51 ± 3.79^{ab}	97.52 ± 3.32^{ab}	87.36 ± 3.29^{bc,***}	76.14 ± 6.20^{abc,***}	84.62
S3	116.17 ± 4.34^{a,***}	108.70 ± 2.2^{a,*}	94.74 ± 2.61^{bc}	84.65 ± 1.45^{c,***}	77.40 ± 1.26^{ab,***}	81.21
S4	88.11 ± 2.36^{f,***}	87.20 ± 3.09^{d,***}	81.92 ± 0.77^{def,***}	73.65 ± 1.39^{f,***}	69.71 ± 2.71^{cde,***}	8.48
S5	106.51 ± 2.48^{bcd}	91.77 ± 3.72^{d,*}	86.52 ± 1.80^{d,***}	79.87 ± 4.38^{d,***}	64.37 ± 2.90^{e,***}	37.22
S6	87.53 ± 0.74^{f,***}	89.12 ± 2.62^{d,***}	84.30 ± 2.72^{de,***}	75.73 ± 1.40^{def,***}	72.21 ± 3.77^{bcd,***}	10.06
S7	98.94 ± 0.93^{e}	87.28 ± 1.32^{d,***}	79.11 ± 2.97^{f,***}	75.00 ± 1.46^{ef,***}	63.42 ± 0.70^{e,***}	17.70
S8	110.24 ± 5.37^{abc,***}	108.58 ± 2.04^{a,*}	97.69 ± 4.43^{ab}	90.12 ± 2.89^{ab,**}	82.55 ± 2.15^{a,***}	106.90
S9	109.62 ± 4.75^{abc,**}	103.77 ± 5.46^{abc}	92.52 ± 3.58^{c}	85.08 ± 1.33^{c,***}	72.18 ± 6.64^{bcd,***}	68.01
S10	100.86 ± 3.15^{de}	99.81 ± 1.35^{bc}	91.68 ± 0.56^{c,*}	75.54 ± 3.80^{def,***}	67.98 ± 1.76^{de,***}	43.10
S11	103.77 ± 4.02^{cde}	88.63 ± 6.25^{d,***}	81.50 ± 1.87^{ef,***}	67.49 ± 2.24^{g,***}	67.11 ± 2.47^{de,***}	19.75
S12	106.86 ± 4.54^{bcd}	98.48 ± 4.27^{c}	86.08 ± 2.82^{de,***}	79.04 ± 1.37^{de,***}	67.73 ± 3.98^{de,***}	42.33

Note: Data were presented as percentage of absorbance ratio between sample-treated groups and control groups, and as means ± standard deviations (n = 3). The cell viability cells at different treated concentration were marked separately by different letters with significant difference ($p < 0.05$). *, $p < 0.05$; **, $p < 0.01$; ***, $p < 0.001$ were labeled versus vehicle control which was defined as 100%.
